# Nitrogen Use Efficiency Definitions of Today and Tomorrow

**DOI:** 10.3389/fpls.2021.637108

**Published:** 2021-06-04

**Authors:** Kate A. Congreves, Olivia Otchere, Daphnée Ferland, Soudeh Farzadfar, Shanay Williams, Melissa M. Arcand

**Affiliations:** ^1^Department of Plant Sciences, University of Saskatchewan, Saskatoon, SK, Canada; ^2^Department of Soil Science, University of Saskatchewan, Saskatoon, SK, Canada

**Keywords:** NUE indices, fertilizer, nitrogen cycling, agroecosystems, sustainability metrics

## Abstract

Crop production has a large impact on the nitrogen (N) cycle, with consequences to climate, environment, and public health. Designing better N management will require indicators that accurately reflect the complexities of N cycling and provide biological meaning. Nitrogen use efficiency (NUE) is an established metric used to benchmark N management. There are numerous approaches to calculate NUE, but it is difficult to find an authoritative resource that collates the various NUE indices and systematically identifies their assets and shortcomings. Furthermore, there is reason to question the usefulness of many traditional NUE formulations, and to consider factors to improve the conceptualization of NUE for future use. As a resource for agricultural researchers and students, here we present a comprehensive list of NUE indices and discuss their functions, strengths, and limitations. We also suggest several factors—which are currently ignored in traditional NUE indices—that will improve the conceptualization of NUE, such as: accounting for a wider range of soil N forms, considering how plants mediate their response to the soil N status, including the below-ground/root N pools, capturing the synchrony between available N and plant N demand, blending agronomic performance with ecosystem functioning, and affirming the biological meaning of NUE.

## Introduction

Reactive N has been identified as one of the top five emerging threats facing humanity and the planet (UNEP, 2019) due to its impact on the climate, environment, and public health—and is attributed to the global reliance on reactive N for food production (Erisman et al., 2008, 2011). Consequently, governing leaders are adopting sustainable N management resolutions (UNEA-4, 2019). To ensure the success of such resolutions, accurate and meaningful N metrics are needed in order to identify, monitor, and develop management practices or innovations that reduce N pollution. One commonly used metric is N use efficiency (NUE); as it can be used for environmental *and* economic objectives of minimizing nutrient losses and the negative impact on surrounding water, air and ecosystems, as well as reducing costs associated with excessive fertilizer inputs (Galloway et al., 2014).

There have been many scientific contributions on the topic of NUE, predominately focused on how to improve crop NUE via agricultural management or breeding innovations (Moll et al., 1982; Raun and Johnson, 1999; Cassman et al., 2002; Cormier et al., 2016; Martinez-Feria et al., 2018). This body of literature encompasses a wide range of NUE calculations, and acknowledges that different NUE indices have distinctive functions (Good et al., 2004; Ladha et al., 2005; Dobermann, 2007; Fageria et al., 2008; Ernst et al., 2020). Key reviews have collated several common measures of NUE and their recommended applications (Dobermann, 2007; Fixen et al., 2015); however, it is difficult to find a single authoritative source that presents a comprehensive list of all NUE indices, and a systematic comparison of their strengths and limitations. Such a resource would provide researchers and students with a tool to critically analyze NUE and decide which indices are best used depending on the research question or circumstance.

At first glance, NUE may appear as a simple term and concept, but its complexity resides in the various N sources that contribute to crop production (inorganic and organic fertilizers, soil organic matter, biological fixation, atmospheric deposition); the interplay between soil N availability, transformation, storage, movement and loss; edaphic conditions; crop genetics; and the impact of management, weather, and climate. A solid understanding of the controlling factors, spatial and temporal boundaries, and intended end-use is needed when interpreting NUE results. To add to this complexity, there is reason to question the usefulness of many conventional NUE indices and whether or not the way that NUE is conceptualized, yet alone computed, should be reconsidered (Erisman et al., 2018; Martinez-Feria et al., 2018). Most conventional NUE indices are principally focused on evaluating crop responses to N or N balance, and operate on a short-term scale (Dobermann, 2007; Fixen et al., 2015). This interpretation of NUE does not express how tightly N is cycled over time within cropping systems (in other words, how prone a system is to N loss), and prioritizes yield at the risk of mischaracterizing the fate of N (Martinez-Feria et al., 2018). Future NUE metrics must capture N cycling to help meet the goals of designing sustainable agricultural systems—a multifaceted objective that not only considers crop production but also the sustainability of soil fertility and mitigation of environmental pollution. To reconcile and deliberate various interpretations of NUE, our objectives are to methodically assess various NUE indices and their definitions, identify their strengths and limitations, and to discuss prospective improvements to the conceptualization of NUE.

## Nitrogen Use Efficiency Indices Commonly Used in Agricultural Research

The multitude of NUE indices commonly used in agricultural research are categorized into groups by denominator, such as: fertilizer-based, plant-based, soil-based; also, by approach: isotope-based, or systems-based NUEs (Table 1). The intended research question(s) dictate which NUE indices are selected and employed, but no NUE index is without weakness (Table 1). As such, it is often recommended to use multiple NUE approaches to ensure the representation of different insights (Van Eerd, 2007; Fixen et al., 2015).

**TABLE 1:** Various NUE indices and their associated formulae^a^, interpretation, strengths, and limitations.

NUE Indicator	Formula	Interpretation	Strengths	Limitations	Key References
***Fertilizer-based***					
Partial-factor Productivity (PFP)	$=\frac{{\mathrm{Yield}}_{\text{f}}}{\mathrm{Fertilizer}N}$	The expression of yield per unit of fertilizer N applied	- Grower-friendly due to simple numerator and denominator - Best for comparing management practices when focusing on a single crop type - Allows estimation at both regional or national levels if records of inputs and outputs are available	- Does not account for background (indigenous) soil N - Cross-site comparisons are limited by neglecting to account for background soil N - Limited meaning on non-N responsive soils - Risk of overestimating NUE under conditions where soil N has built up	Dobermann (2007)
N Balance Intensity (NBI)	= *Yield N* − *F ertilizer N*	The difference between fertilizer N applied and the N removed as yield; commonly called N surplus	- Shows if there is an accumulation or decline in soil N over a predetermined timeframe (i.e., growing season) - The closer the difference is to zero, the smaller the accumulation of N in the system - Positive values likely reflect a decline in the soil fertility	- Does not account for background soil N	IPNI (2014)
NUE_crop_	$=\frac{YieldN_{f}}{FertilizerN}$	The fraction of fertilizer N that is utilized and allocated to yield N	- Values greater and less than 1 indicates net removal and surplus of N, respectively	- Does not account for background (indigenous) soil N or mineralized N during the growth season	Martinez-Feria et al. (2018) but their denominator included total N inputs
Partial N Balance (PNB)	$=\frac{PlantN_{f}}{FertilizerN}$	The expression of plant N content per unit of fertilizer N applied	- Values > 1 indicate soil mining; whereas values < 1 indicate excessive N application	- Does not account for background (indigenous) soil N - Highly changeable NUE in short-term trials due to fluctuations in soil N via mineralization and immobilization N	Dobermann (2007)
Agronomic Efficiency (AE)	$=\frac{Yield_{f}-Yield_{0}}{\mathrm{Fertilizer}N}$ = PE × RE	The contribution of fertilizer N towards yield, compared to a non-fertilized control	- Useful when focusing on the economic portion of plants (yield) - Indicates the relative benefit of fertilizer to soil N	- Short-term trials can underestimate AE by neglecting residual effects of repeated fertilizer applications - Not suitable for trials without non-fertilized control plots	Dobermann (2007)
Fertilizer-N Recovery Efficiency (RE_fertN_)	$=\frac{PlantN_{f}-PlantN_{0}}{\mathrm{Fertilizer}N}\times 100$	The percentage of fertilizer N that is taken up by the plant, accounting for background soil N levels; also sometimes referred to as apparent recovery	- Accounts for background soil N Useful in looking at crop response to applied fertilizer	- Challenging to use in long-term trials if soil N reserves are depleted in the non-fertilized control treatment - Only useful in trials with non-fertilized control plots	Dobermann (2007)
***Plant-based***					
Physiological Efficiency (PE)	$=\frac{Yield_{f}-Yield_{0}}{\mathrm{Plant}N_{f}-PlantN_{0}}$	The contribution of fertilizer N from the plant tissues towards the yield component	- Accounts for background soil N - Useful to identify plants that have a superior ability in producing yield per unit of available N	- Not suitable for long-term trials, as depletion of indigenous soil N in non-fertilized controls can lead to erroneously high NUEs - Not suitable for trials without non-fertilized control plots	Dobermann (2007)
N Utilization Efficiency (NUtE)	$=\frac{Yield}{PlantN}$	Similar to PE, but does not account for background N	- Useful to identify plants that have a superior ability in producing yield relative to plant tissue N	- Does not account for background soil N	Moll et al. (1982)
Internal Efficiency (IE)	$=\frac{YieldN_{f}}{PlantN_{f}}$	The fraction of plant tissue N that is contained in the yield component	- Useful to identify plants with high N translocation to the economic component (yield)	- Does not account for background soil N - Results confounded with soil nutrient status, i.e., high IE values may indicate N deficiency rather than increased NUE	Dobermann (2007)
N Harvest Index (NHI)	$=\frac{YieldN}{PlantN}\times 100$	The same as IE, but expressed as a percentage	- Useful to identify plants with high N translocation to the economic component (yield)	- Does not account for background soil N - Results confounded with soil nutrient status, i.e., high NHI values may indicate N deficiency rather than increased NUE	Moll et al. (1982)
NUE_soil_	$=\frac{Plant}{\mathrm{Fertilizer}N+\mathrm{Soil}N}$	The biomass production per unit of available N	- Useful to identify crops with potential to produce large amounts of biomass per unit of available N	- Soil inorganic N measurements usually collected prior to planting, thus only captures a snapshot of the N that is available throughout the season - Does not account for how N is cycled in a system - Limited applicability at large spatial and temporal scales, as crop response to soil N may be influenced by other factors	Moll et al. (1982)
***Soil-based***					
N Uptake Efficiency (NUpE)	$=\frac{PlantN}{\mathrm{Fertilizer}N+\mathrm{Soil}N}\times 100$	The percentage of available soil N that is utilized by the plant; also conceptualized as apparent recovery efficiency of the N supply	- Can point towards improved synchrony between N availability and plant demand	- Without having access to a non-fertilized control, this does not account for soil N mineralized throughout the growing season	Moll et al. (1982)
NUE_yield_	= NUpE × NUtE	The contribution of N supplied from the soil that is allocated to the yield N; also often referred to as simply NUE	- Enables comparisons of yield potential among crop genotypes	- Does not allow comparison between farms, as soil and environmental factors may mask differences in NUE - Yield potential may be influenced by poor soil fertility - Poor N remobilization from vegetative parts to grain adversely impact yield	Novoa and Loomis (1981)
NUE_balance_	$=\frac{Noutputs}{Ninputs}$	The fraction of N inputs that are removed from the system (either as yield or N losses)	- Indicates whether the soil is a net sink (< 1) or a net source (> 1) of N - When the value approaches unity, then the soil pool can be thought of as in equilibrium	Spatial and temporal boundaries can be arbitrary and nebulous - If N data is unavailable, then assumptions are heavily relied upon (having all N losses measured over time is rare)	Martinez-Feria et al. (2018), but they referred to it as NUE_soil_
***Isotope-based***					
N derived from Fertilizer (NdfF)	=N15atom%excessinplantorsoilN15atom%excessoffertilizerN×100	The percentage of plant or soil N that is derived from the fertilizer	- Useful in tracing the relative proportion of fertilizer N taken up by the crop, separate from soil N sources	- ^15^N technique can be expensive - Relies on meticulous sampling and measurement techniques	IAEA (1983)
Total N derived from Fertilizer (TNdff)	= $\frac{Ndff}{100}\times$ *Plant N or Soil N*	The total quantity of plant or soil N that is derived from fertilizer	- Useful in determining the quantity of fertilizer N taken up by the crop, separate from soil N sources	- as above	IAEA (1983)
Recovery Efficiency of N-Fertilizer (^15^NRE)	= $\frac{TNdffinPlantorSoil}{FertilizerNapplied}\times 100$	The percent recovery, or utilization, of fertilizer-N in plant and/or soil components	- Directly measures the recovery efficiency of fertilizer N into plant and soil components	- as above	IAEA (1983)
***Ecology-based***					
Nitrogen Productivity (NP)	$=\frac{RelativeGrowthRate}{\left[PlantN\right]}$	The ratio of the relative growth rate to the concentration of N in plant tissues	- Provides a snapshot in time of the plant’s immediate NUE	- Less helpful in providing insight into the plant’s long-term or potential performance	Berendse and Aerts (1987)
NUE_*ecology*_	= NP × MRT	The product of N productivity and the mean residency time (MRT) of plant N	- Considers the temporal dimension of NUE, the average time that N remains in the plant before it is lost or shed	- Not originally intended for agricultural systems where some crops are harvested/used beyond physiological maturity	Lambers and Oliveira (2019)
***System-based***					
N Balance Index of a System (sNBI)	= *N input* − *N ouput* − Δ*soil total N*	The accumulation or reduction of soil N over a set time	- Indicates an accumulation or decline in soil N over a predetermined timeframe (i.e., growing season) - Accounts for more sources, sinks, and N losses than NBI, i.e., soil N pools in addition to fertilizer N as inputs, and considers more than just yield (i.e., N losses) as N outputs - Useful at various scales	- Boundaries can be arbitrary or nebulous If N data is unavailable, then assumptions are heavily relied upon - Meaning is strongly influenced by a system’s sensitivity to N loss - The balance approach provides limited nuance for the dynamics of N cycling and recycling	Sainju (2017)
NUE of a System (sNUE)	$=\frac{YieldN}{YieldN+NLoss}$	The fraction of system N outputs that are captured as N yield rather than lost to the environment	- Considers the link between crop and soil N - Higher values indicate tighter the N cycling (greater recycling); lower values indicate N release to the environment (leaking)	- Strongly influenced by soil conditions and background soil fertility	Martinez-Feria et al. (2018)
NUE of Food Chain (NUE_FC_)	= $\frac{Navailableforconsumption}{NewNInput}$	The N balance of the entire food chain system, in terms of N consumed as protein relative to N inputs	- Provides an estimate for the amount of N converted to food protein for consumption - Encompasses all N inputs, use, outputs and losses from production to consumption - Useful in both plant and animal production systems - Detects systems with declining efficiency and opportunities to improve NUE - When records are available, allows estimation at larger scales, from field to continental	- Lack of a universal method or approach for its estimation - High degree of uncertainty	Erisman et al. (2018)
Virtual N factor (VNF)	= $\frac{Nusedtoproducefooditemthatendsuprecycled}{Ninfooditemthatisconsumed}$	The portion of the N that is released to the environment during the food production process and is not contained in the food that is consumed	- Virtual N considers N losses such as fertilizer runoff, processing wastes, manure losses, and food waste	- High degree of variability across regions due to differences in diets and transportation of food items	Galloway et al. (2014)

*^*a*^Formula variable descriptions: Yield and Plant variables followed by subscript *_*f*_* specifies fertilized conditions, whereas subscript *_0_* specifies non-fertilized conditions. For example, Plant N_*f*_ denotes the amount of N in a fertilized plant; Plant_*f*_ denotes the weight of a fertilized plant. Fertilizer N is conceptualized as the inorganic N contained in any form of N input (from synthetic or organic sources). Soil N is measured as soil inorganic N levels. While this paper is focused on N, these indices could similarly be applied to other nutrients.*

### Temporal and Spatial Boundaries of NUE Indices

Interpretation of the NUE indices depends on the spatial and temporal boundaries to which the indices are applied. Calculations of NUE based on a single growing season can provide valuable crop- and site-specific information but limit the inferences that can made across a crop rotation or about the performance of the cropping system or N cycling over time. In general, most fertilizer- and plant-based indices are suited to (or originally created to address) short-term time scales such as growing seasons; likewise, for many soil-based or isotope-based indices—however, these can also be applied at longer-term scales such as multi-year crop rotations. Ecology-based indices generally apply to a plant’s life cycle, whereas system-based indices tend to have a more flexible time-scale selection, depending on the goals of the user.

Most NUE indices rely on ratios or proportions of crop yield or N *vs*. soil and fertilizer N sources within the spatial bounds of a single field or experimental plots. Indices based on N balances, however, require more refined definitions of spatial boundaries to account for N inputs to outputs—thus it could be applied at the field scale, or applied to a farm, watershed, regional, or global scales. In addition to defining boundaries, defining what a “system” is and where its boundaries lie can be both arbitrary and nebulous, generally introducing higher degrees of uncertainty as the bounds of the “system” are expanded. Common boundaries for NUE range from a single plant, a field, a farm, watersheds, or regions.

### Fertilizer-Based Indices

Fertilizer-based NUEs express the amount of fertilizer applied relative to various plant parameters, such as aboveground biomass, yield or N content. Agronomic efficiency (AE), partial factor productivity (PFP), and N balance intensity (NBI) focus on the economic portion of plants (the crop yield), and this agronomic perspective is useful for deciphering how to increase or maintain crop yield while minimizing inputs. The recovery efficiency of fertilizer (RE_fertN_) illustrates the apparent increase in plant N uptake in response to the N input, whereas the AE addresses how much productivity is improved by the application of N. The PFP targets the question “how productive is the cropping system relative to the N input?” The partial N balance (PNB) reports the ratio of N removal to N use, and the NBI addresses the difference between N removed and N used. The NUE_crop_ addresses the proportion of yield N relative to N input. Generally, fertilizer-based indices are useful for computing the most economical rate of N fertilizer (MERN), when used in combination with dose response curves.

Calculating fertilizer-based NUEs for a single growing season will not necessarily provide the same results if considering fertilizer-based NUEs for a cropping system (or long-term time scale). When calculated from annual response data, certain fertilizer-based NUEs—namely RE_fertN_ and AE—will produce different estimates of NUE than if these indices were calculated over a longer-term time scale, if the system is not in equilibrium. For example, if the system has a surplus of N relative to crop demand, then neglecting to account for residual fertilizer N or repeated fertilizer applications over the years would underestimate NUE. Understanding annual responses in addition to long-term nutrient dynamics and crop NUE are central agronomic questions.

Fertilizer-based NUE indices that ignore soil N supply (PFP, PNB, and NBI) may produce inconsistent results because of the variation in soil inorganic N supply. For example, if the soil is rich in mineralizable N, then favorable crop yields may be achieved without applying N fertilizer. In this situation, a small amount of N fertilizer would lead to a very high fertilizer-based NUE; correspondingly, larger amounts of N fertilizer would decrease the fertilizer-based NUE. In a case such as this, a “low” fertilizer-based NUE demonstrates that the background soil N supply meets or exceeds plant demand, rather than demonstrating that a plant uses the available N “less efficiently”. Fertilizer-based NUEs are therefore useful to identify situations of N saturation or deprivation, more than any other interpretation about a plant’s ability to use the N available to it. Despite the gross simplicity, indices like PFP remain popular in agronomic advisory contexts because producers know the terms (yield and N applied) and have control over the latter.

Of the fertilizer-based indices, only RE and AE consider background soil N levels by accounting for N uptake or production in non-fertilized control plots; as such, both are relevant for research contexts. While this is better than altogether ignoring the impact of background N levels on the crop N response (as done in PFP, PNB, and NBI), it can still provide misleading NUEs if the soil N reserves in the non-fertilized control plots are depleted over long-term periods. Further, the formulation overlooks the reality that plants mediate their response to N and have multiple strategies to cope with N limitation by shaping and recruiting N-cycling microbial communities (Moreau et al., 2019)—as discussed later.

### Plant-Based Indices

Plant-based indices focus on the allocation of plant tissue N towards crop yield or yield N (Table 1), providing information that is not provided by fertilizer-based NUE indices. Plant-based indices are useful for identifying plant genotypes with enhanced capability of allocating growth or N resources towards the economic portion of plants—thus useful for breeding. The physiological efficiency (PE) illustrates the ability of a plant to transform N acquired from fertilizer into economic yield. The N utilization efficiency (NUtE) addresses the yield produced per unit of N acquired by the plant shoots. The internal efficiency (IE) and N harvest index (NHI) (essentially the same index, only one is a fraction and the other is a percentage) relate the N allocated to yield relative to the whole plant N.

By using IE and NHI, one can indirectly determine the amount of N that is returned to the soil after harvest in the form of crop residue-N (the plant tissue N that was not removed with yield from the field). Residue-N serves as an N source to subsequent crops, depending on the timing of mineralization relative to plant N demand. However, it may also be at risk for N loss, or stored in the soil in various forms of inorganic and organic N. The information in IE and NHI about N returned to the soil after harvest can be useful to environmental specialists, as well as for crop residue management.

Crop traits and characteristics that favor N mobilization and translocation toward the reproductive organ will strongly influence the interpretation of results; hence, genetic and physiological factors are key determinants of plant-based NUE results. As a shortcoming, plant-based NUE indices can output misleadingly high values due to the depletion of indigenous soil N (i.e., long-term non-fertilized controls) or N deficiency, rather than an intrinsic capability for improved NUE. For this reason, results can be ambiguous. When defining NUE as a ratio of yield or yield-N to aboveground plant tissue or plant tissue-N, this perspective leaves out an important N pool: belowground and root N contributions—discussed later.

### Soil-Based Indices

Soil-based indices not only account for fertilizer N inputs but also the soil N contributions to the plant components (Table 1). Consequently, the results and interpretation are primarily influenced by soil management and N dynamics. These indices are intended to capture soil inorganic N availability and the mineralizable N made available over the growing season for crop production—quantities that can be best estimated when non-fertilized controls are included. The use of non-fertilized controls carries some assumptions about the supply of N from soil sources, including that N fertilizer addition does not affect soil mineralization and immobilization processes and that there are no losses of any available soil N from the control plots (Huggins and Pan, 1993). Huggins and Pan (1993) also point out that because the control plots provide knowledge on the amount of *net* N mineralization (not *gross* N mineralization), N made available through mineralization is inherently underestimated.

The NUE_soil_ reports the plant biomass accumulation per unit of soil N available, whereas N uptake efficiency (NUpE) elucidates the capture of available N by plant roots and subsequently utilized by the plant. The NUpE can also be conceptualized as the apparent recovery efficiency of available soil N in the plant. The product of NUpE and NUtE is NUE_yield_ (often referred to as NUE) but because the biomass strongly depends on the N taken up, uptake and utilization efficiency are not independent of each other. The NUE_balance_ indicates whether the soil is a net source (>1) or sink (<1) of N.

By accounting for N outputs relative to available N (i.e., NUpE and NUE_yield_), one can indirectly estimate the risk for N losses—theoretically, any N that is not removed after harvest or that is unused by the plant at the end of the growing season could be subjected to loss. Still, this interpretation does not quantify how tightly N is cycled, nor does it differentiate the balance between soil N storage and N loss. Therefore, a reliable metric of losses would only be attained if longer-term scales are considered, and where processes like immobilization-mineralization are in equilibrium.

### Isotope-Based Indices

Nitrogen-15 labeling techniques are used to trace the flow of N derived from soil, fertilizer, or biologically fixed N within cropping systems; they can provide quantitative data on pool sizes and movement of N along the soil-water-plant-atmosphere continuum. With the use of ^15^N-labeled fertilizers, researchers can directly measure fertilizer recovery into plant components (^15^NRE) and determine the proportion and total amount of N derived from fertilizer (NdfF) in a single growing season. In knowing the proportion of NdfF, one can also determine the proportion of N attributed to indigenous soil N. Unlike the N difference methods used in the fertilizer- or soil-based indices (where N is expressed as a percentage or fraction of fertilizer N applied or soil available N), the ^15^N method provides a direct measurement of recovery, as opposed to an apparent recovery. Further, other ^15^N labeled sources of N can be studied; for example, not just fertilizer N but crop residue-N or soil N sources. Multi-year ^15^N monitoring can be designed to provide information on the longer-term use and recovery of N, to understand soil-plant N cycling, and the N contribution to plants from biological N fixation, plant roots, crop residues, and indigenous soil N (Meier et al., 2006; Arcand et al., 2014; Taveira et al., 2020).

### Ecology-Based Indices

More than 30 years ago, Berendse and Aerts (1987) argued that a biologically meaningful definition of NUE should include two components (1) the period during which the absorbed N can be used for C fixation, per unit of N in the plant and (2) the instantaneous rate of C fixation per unit of N in the plant. They proposed a NUE index which combined N productivity (NP) with mean residency time; this conceptualization of NUE describes the dry weight which can be produced per unit of N taken up, under steady-state conditions (but steady-state N dynamics, where N inputs are balanced by N losses, may not always be the case for agricultural systems).

Nitrogen productivity is the ratio of relative growth rate (RGR) to the whole plant nutrient concentration in the tissues (PNC; Table 1). Higher NP is associated with rapid growth, a relatively large investment of N in photosynthesizing tissue, an efficient use of N invested in the leaves for the process of photosynthesis, and a relatively small use of carbon in respiration—as explained by Lambers and Oliveira (2019). Although the NP provides a meaningful clue of a plant’s immediate NUE, it is less helpful in providing insight into the plant’s long-term or potential performance in agroecosystems. To achieve this, Lambers and Oliveira (2019) expand the concept of NUE to consider time, as earlier suggested by Berendse and Aerts (1987). Plant NP is integrated with mean residence time (MRT) of N in the plant (NP x MRT). In this view, the mean residence time is the average time that N remains in the plant, before it is lost due to leaf shedding, herbivory, root death, etc.

### Systems-Based Indices

System-based NUE indices—defined by spatial and temporal boundaries—are formulated as a balance or a difference (Table 1). For sNBI or sNUE, crop- and soil-based approaches are linked to encompass multiple parts of system performance (Table 1). A system-approach enables comparisons that differ in soil properties, crop sequences, climate, etc., thereby capturing differences in biophysical controls on N dynamics (Martinez-Feria et al., 2018). The sNUE index can be used to identify systems that tightly cycle N or that release N to the environment. By including yield N as a numerator for sNUE, it allows for examining trade-offs between N flows (in and out) when compared to other factors like crop production.

As a step further, NUE indicators can be computed for the entire food chain system (NUE_FC_), defined by the series of processes by which food is produced and eventually consumed. This information is useful for computing N-footprints and developing information tools for consumers and institutions (Leach et al., 2012), and developing N-labels or N loss indicators as a decision tool for consumers (Galloway et al., 2014). Researchers have defined NUE_FC_ as the ratio of the protein (expressed as N) available for human consumption to the N input (newly fixed and imported) to the food system, but it is acknowledged that the final NUE_FC_ value may have considerable variability and uncertainty (Erisman et al., 2018). The virtual N factor (VNF) is another type of “food chain NUE,” which estimates the proportion of N that is not consumed but was either released to the environment or wasted. Virtual N includes fertilizer runoff, processing wastes, manure losses, and food waste, and VNFs relate the virtual N lost to the food consumed. There can be wide degree of variability in VNFs due to differences in the diet, particularly with respect to the relative amounts of plant versus animal protein consumption, how food is produced, and its transportation from its source to the consumers (Galloway et al., 2014).

## The Nue Indices of the Future

Nitrogen use efficiency is regulated by biological, physiological, environmental, genetic, agronomic, and developmental factors; thus, a multi-disciplinary approach that encompasses several factors is essential for improving the veracity of NUE indices (Hirel et al., 2011). As outlined above, there are several limitations in the way that current NUE indices are conceptualized. In contemplating how to advance NUE as a biologically meaningful index, here we pose several questions: Are all forms of biologically available N being adequately considered in current NUE indices, and if not, what other forms should be? How might NUE indices better represent the plant’s role in modulating its response to N? Can NUE move beyond accounting for inputs and outputs, towards considering the temporal and spatial synchrony of N availability and plant N demand? Perhaps integrating components of ecosystem functioning into the conceptualization of NUE would improve the meaning of such indices? While no single measure of NUE will satisfy all concerns, contemplating questions such as these is intended to promote the advancement of how NUE is theorized.

### Considering Forms of N Other Than Nitrate and Ammonium

A conspicuous oversight in how several NUE indices are conceptualized is the neglect to consider forms of N other than inorganic N (with the exception of the soil-based formulas that include unfertilized controls and/or capture background N contributions). Historic and recent discoveries demonstrate that plants take up N directly as organic molecules, such as amino acids, peptides, and even proteins (Rentsch et al., 2007; Näsholm et al., 2009; Hill et al., 2011; Paungfoo-Lonhienne et al., 2012; Dion et al., 2018; Enggrob et al., 2019) and that plants capitalize on carbon already contained in organic N sources, thereby improving NUE (Franklin et al., 2017). This phenomenon is a promising new area, largely enabled by compound-specific stable isotope tracking, that will likely move us towards an improved understanding of NUE in cropping systems (Farzadfar et al., 2021). Future NUE formulations should consider the organic N sources that contribute to plant growth and production.

To better capture all the forms of N that plants use, the reconceptualizing of NUE must go beyond simply adjusting the denominator to include the soil organic N pool that is available to and usable by plants—as this would numerically reduce several traditional estimates of NUE. Rather, a highly N efficient plant would be, theoretically, one that is capable of using the forms of N biologically available to it, and which adjusts the relative proportion of N taken up in each form based on minimizing its energy expenditure during the process. As plants exercise the ability to take up a suite of N forms in an energy-dependent manner, a highly efficient agroecosystem would be one that achieves a balance between N saturation and deprivation—as saturation would risk N loss, whereas deprivation would risk crop productivity.

One example for how novel NUE indices might be formulated is: NUE_bio_ = 1 – (plant N uptake capacity/soil bioavailable N supply), where NUE_bio_ results approaching zero would signify scenarios with higher efficiency. Positive values would signify a soil N surplus relative to plant capacity, whereas negative values would signify a soil N deficit relative to plant capacity. The soil bioavailable N supply would encompass inorganic and organic forms of N that plants are capable of taking up; the plant uptake N capacity would reflect the plant’s functional demand for N. This, of course, would require accurate measurements of plant N uptake capacity (and demand) and soil bioavailable N supply—a perhaps lofty but worthwhile ambition for future researchers.

### Accounting for the Synchrony Between N Availability and Plant N Demand

The synchrony of crop N demand relative to soil available N is not captured in most NUE indices, even over short-term periods like a single growing season. The fluctuation in soil available N throughout a single growing season can be considerable. Even if the soil test-N shows substantial amounts of available N at planting, this does not guarantee sufficient available N when the crop is rapidly growing and requires N. If the N is lost or unavailable by the time that the plant is actively taking up N, then the NUE results would not truly reflect the plant’s “efficiency” of taking up available N. Improved NUE indices should consider the *timing* and dynamic *fluctuation* of plant N uptake relative to soil available N. Perhaps a “synchrony factor” will be a part of futuristic NUE indices. In theory, the most N efficient scenarios would be realized when the soil N supply matches the plant’s demand for N to meet its functional requirements at all times throughout the plant’s growth cycle, and this might be conceptualized by considering NUE as an *integral*. For example, if NUE_bio_ was plotted on a y-axis and time was plotted on the x-axis, one could compute the *net* area under the curve to determine if the plant experiences a net surplus of bioavailable N or deficit relative to its needs throughout the growth cycle. This might look something like $\lim_{n\to \infty }NUE_{bio_{n}}=\int_{x_{0}}^{x_{n}}f\left(x\right)dx$ and defined as the net area between the NUE_bio_ function over the interval of time studied. Resulting *net* values that approach zero would indicate that, over the plant growth period, there was a balance between soil bioavailable N supply and plant N capacity. Scenarios that give rise to a balance between soil bioavailable N and plant N capacity would, arguably, be the most efficient at using N, and support tighter N cycling. In practice, collecting the necessary measurements to compute the integral for NUE_bio_ would be very challenging, as it would entail knowing the soil bioavailable N supply and the plant N uptake capacity almost continuously throughout a plant’s growth cycle (or at least very frequently, so that the area under the curve could be best approximated). Nonetheless, ideas such as this are put forth for the purpose of spurring advancements about how NUE can be better conceptualized in the future.

### Representing How Plants Mediate Their Response to N

Although not represented by current NUE indices, plants regulate their response to soil N via multiple strategies, such as shaping and recruiting N-cycling microbial communities (Moreau et al., 2019), manipulating microbial functional groups (Whipps, 2001; Fontaine et al., 2007; Blagodatskaya et al., 2009), and modifying their capability to compete with bacteria for N (Legay et al., 2020). Plants are responsive to the soil nutrient status and will modify their root system architecture and morphology accordingly, which in turn modifies the volume of soil accessed and influences soil N mineralization (Hodge, 2004). As for root N uptake, the mode of uptake (and therefore efficiency of acquisition) will depend not only on external inorganic N concentrations but the organisms delivering N-compounds—categories that are not currently captured by the NUE indices listed in Table 1.

Plant symbioses with arbuscular mycorrhizal fungi (AMF) can improve N uptake and affect NUE (Verzeaux et al., 2017). Arbuscular mycorrhizal fungi associations can be enhanced under low soil N conditions, expanding the root surface area to explore a larger soil volume, accelerate N mineralization, and facilitate uptake of N from organic N sources (Thirkell et al., 2016). While there is doubt that AMF can play much of a role in improving NUE in high fertility agricultural soils (Garnett et al., 2009), AMF may be important even in soils with high N availability where the fungal partners are more efficient at proliferating and acquiring NH_4_^+^ in N-rich patches than plant roots (Hodge and Fitter, 2010; Fitter et al., 2011; Veresoglou et al., 2012). In spite of conflicting reports on exact mechanisms or degree of effects, AMF can affect both soil N cycling and N uptake with potential impacts on NUE.

As another illustrative example: N fixing plants can be thought of as the most efficient N users. If the soil N supply is below the plant N requirements, then N fixing plants expend energy to ensure their own N supply via nodulation and biological N fixation. However, if any one of the existing fertilizer- or soil-based NUE formulations (Table 1) were applied to N fixing plants, the resulting NUE value would provide rather limited information about how these plants use N in a biologically meaningful way. This example demonstrates how the traditional NUE formulations can fall short of reporting a meaningful NUE value, and where reconceptualizing NUE is needed. Further, NUE indices could be improved to express the plant physiological functioning of N, rather than simple N mass balance or allocation.

### Including Root N Pools and Considering the Rhizosphere

For a more complete picture of measuring NUE, the entire plant should be considered—including the roots. Roots comprise roughly 14% of total plant N in common annual crops (Gan et al., 2010), with 4–71% of total plant N released to soil as rhizodeposits (Wichern et al., 2008). Considering how plants allocate N to roots will affect all NUE metrics that include plant N (Table 1). By ignoring root components, some metrics may underestimate NUE (e.g., NUpE, NUE_soil_) whereas others may overestimate NUE (e.g., PE, NUtE, NHI). Root N levels can serve as an internal N source for reproductive organs as the plant’s life cycle progresses. Accurately measuring the amount of N that is withdrawn from the senescing root parts is difficult, but it is estimated that 50% of N in dying roots is withdrawn and redistributed (Berendse and Aerts, 1987)—a phenomenon that undoubtedly influences overall plant NUE. For a more biologically meaningful definition of NUE, plant N response strategies and translocation dynamics from roots should be considered.

Including root components and rhizosphere dynamics in NUE indices is not without challenges, as many studies do not measure roots and rhizosphere components. However, techniques to measure root traits and rhizosphere dynamics are advancing in the plant and soil sciences, offering future opportunity to better address belowground influences on NUE. If direct measurements are not available, then assumptions will be relied upon. Nonetheless, advanced conceptualizations of NUE would be remiss to entirely ignore plant roots and rhizosphere.

### Expressing Agronomic- and Ecosystem-Functioning Aspects of NUE

As the importance and awareness of agricultural sustainability grows, the goals of improving crop production *and* environmental quality are converging. Accordingly, new NUE indices (or a combination thereof) should capture aspects of agronomic performance as well as ecosystem function. Rather than only focusing on what happens to N during the growing season, it is crucial that metrics consider the ‘tightening’ of N cycling (for example, with diversifying cropping systems or N scavenging by cover crops). This might be achieved by linking soil- and plant-based approaches to derive systems-based approaches (Martinez-Feria et al., 2018). Another approach considers the whole life-cycle of a crop (Weih et al., 2011), N carry-over and conservation over more than a single growing season (Dawson et al., 2008). Further, linking ecosystem functioning indices to soil- and plant-based NUE approaches would provide a more holistic picture of NUE. It is possible that “cycling factors” or a life-cycle approach could be developed and integrated into NUE indices one way or another. In merging agronomic and ecological perspectives of NUE, a highly efficient system might be conceptualized as one with minimal to no “N waste,” either in terms of wasted N fertilizer not taken up or wasted N via N losses.

## Conclusion

Meaningful metrics are critical to progress towards meeting the goals of productivity and sustainability. The choice of metric significantly affects the interpretations and conclusions about the performance of the crop or system—and for NUE, there are many. The definitions, strengths, and limitations of the various NUE indices are presented herein to clarify their potential use when addressing different research questions and goals. To advance the biological meaning of NUE, we propose that future NUE indices should consider a wider range of soil N forms, how plants mediate their response to the soil N status, below-ground/root N pools, and the synchrony between available N and plant N demand. Research must endeavor to blend agronomic performance with ecosystem functioning to advance the conceptualization of NUE.

## Data Availability Statement

The original contributions presented in the study are included in the article, further inquiries can be directed to the corresponding author.

## Author Contributions

KC conceived of, wrote, and reviewed the manuscript with contributions from MA. OO and DF drafted the table. OO, SF, and SW made written contributions to certain sections. All authors contributed to the article and approved the submitted version.

## Conflict of Interest

The authors declare that the research was conducted in the absence of any commercial or financial relationships that could be construed as a potential conflict of interest.
